# Deep learning-based multi-modal data integration enhancing breast cancer disease-free survival prediction

**DOI:** 10.1093/pcmedi/pbae012

**Published:** 2024-05-29

**Authors:** Zehua Wang, Ruichong Lin, Yanchun Li, Jin Zeng, Yongjian Chen, Wenhao Ouyang, Han Li, Xueyan Jia, Zijia Lai, Yunfang Yu, Herui Yao, Weifeng Su

**Affiliations:** Guangdong Key Laboratory of Cross-Application of Data Science and Technology, Beijing Normal University-Hong Kong Baptist University United International College, Zhuhai 519087, China; Faculty of Innovation Engineering, Macau University of Science and Technology, Taipa, Macao 999078, China; Department of Computer and Information Engineering, Guangzhou Huali College, Guangzhou 511325, China; Department of Pathology, Sun Yat-sen Memorial Hospital, Sun Yat-sen University, Guangzhou 510120, China; Guangzhou National Laboratory, Guangzhou 510005, China; Dermatology and Venereology Division, Department of Medicine Solna, Center for Molecular Medicine, Karolinska Institutet, Stockholm 17177, Sweden; Guangdong Provincial Key Laboratory of Malignant Tumor Epigenetics and Gene Regulation, Department of Medical Oncology, Breast Tumor Centre, Phase I Clinical Trial Centre, Sun Yat-sen Memorial Hospital, Sun Yat-sen University, Guangzhou 510120, China; The Second Clinical Medical College, Southern Medical University, Guangzhou 510515, China; Faculty of Medicine, Macau University of Science and Technology, Taipa, Macao 999078, China; Guangdong Provincial Key Laboratory of Malignant Tumor Epigenetics and Gene Regulation, Department of Medical Oncology, Breast Tumor Centre, Phase I Clinical Trial Centre, Sun Yat-sen Memorial Hospital, Sun Yat-sen University, Guangzhou 510120, China; Guangdong Provincial Key Laboratory of Malignant Tumor Epigenetics and Gene Regulation, Department of Medical Oncology, Breast Tumor Centre, Phase I Clinical Trial Centre, Sun Yat-sen Memorial Hospital, Sun Yat-sen University, Guangzhou 510120, China; Faculty of Medicine, Macau University of Science and Technology, Taipa, Macao 999078, China; Guangdong Provincial Key Laboratory of Malignant Tumor Epigenetics and Gene Regulation, Department of Medical Oncology, Breast Tumor Centre, Phase I Clinical Trial Centre, Sun Yat-sen Memorial Hospital, Sun Yat-sen University, Guangzhou 510120, China; Guangdong Key Laboratory of Cross-Application of Data Science and Technology, Beijing Normal University-Hong Kong Baptist University United International College, Zhuhai 519087, China

**Keywords:** breast cancer, multi-modality, deep learning, pathological, disease-free survival

## Abstract

**Background:**

The prognosis of breast cancer is often unfavorable, emphasizing the need for early metastasis risk detection and accurate treatment predictions. This study aimed to develop a novel multi-modal deep learning model using preoperative data to predict disease-free survival (DFS).

**Methods:**

We retrospectively collected pathology imaging, molecular and clinical data from The Cancer Genome Atlas and one independent institution in China. We developed a novel Deep Learning Clinical Medicine Based Pathological Gene Multi-modal (DeepClinMed-PGM) model for DFS prediction, integrating clinicopathological data with molecular insights. The patients included the training cohort (*n* = 741), internal validation cohort (*n* = 184), and external testing cohort (*n* = 95).

**Result:**

Integrating multi-modal data into the DeepClinMed-PGM model significantly improved area under the receiver operating characteristic curve (AUC) values. In the training cohort, AUC values for 1-, 3-, and 5-year DFS predictions increased to 0.979, 0.957, and 0.871, while in the external testing cohort, the values reached 0.851, 0.878, and 0.938 for 1-, 2-, and 3-year DFS predictions, respectively. The DeepClinMed-PGM's robust discriminative capabilities were consistently evident across various cohorts, including the training cohort [hazard ratio (HR) 0.027, 95% confidence interval (CI) 0.0016–0.046, *P* < 0.0001], the internal validation cohort (HR 0.117, 95% CI 0.041–0.334, *P* < 0.0001), and the external cohort (HR 0.061, 95% CI 0.017–0.218, *P* < 0.0001). Additionally, the DeepClinMed-PGM model demonstrated C-index values of 0.925, 0.823, and 0.864 within the three cohorts, respectively.

**Conclusion:**

This study introduces an approach to breast cancer prognosis, integrating imaging and molecular and clinical data for enhanced predictive accuracy, offering promise for personalized treatment strategies.

## Introduction

Breast cancer remains a formidable global health challenge, ranking as the most common malignancy among women. Despite early and appropriate interventions, ∼30% of breast cancer cases recur and metastasize distantly, resulting in a 5-year survival rate of <23% [1,2]. This stark reality emphasizes the critical need for early detection, a fundamental aspect in combating breast cancer. Traditional clinical predictors, including biomarkers, clinical imaging, and molecular testing, are invaluable but have inherent limitations such as low sensitivity, high costs, limited availability, and the complex issue of intra-patient heterogeneity [3]. This situation necessitates the development of new methods for reliably predicting recurrence risk and survival in postoperative breast cancer patients, aiming to facilitate timely interventions and improve overall prognosis.

Pathological images, particularly when integrated with molecular features and clinical data, play a vital role in the early detection and treatment of breast cancer. An under-explored but promising area in breast cancer research is the integration of multi-modal data types. This approach combines deep learning techniques with pathological image data, genetic features, and clinical information to predict disease-free survival (DFS) [4]. Histopathology, genomics, and transcriptomics demonstrate significant heterogeneity, which greatly influences cancer progression. It is the synergy and validation provided by these diverse modalities that make integrating such varied data types crucial for accurately predicting breast cancer DFS [5, 6].

To tackle this challenge, recent advancements in artificial intelligence (AI) have inaugurated a new era in the medical field, establishing a link with breast cancer characterized by data-driven precision medicine [7]. The integration of AI algorithms with clinical data has the potential to revolutionize breast cancer prognosis, revealing intricate patterns and relationships that traditional methods might miss. AI's broad scope includes applications in breast cancer such as image analysis, disease outbreak identification, and diagnosis. The imminent impact of AI on medical practice promises to enhance the experiences of both medical practitioners and patients, heralding an era of more precise, streamlined, and universally accessible healthcare [8, 9]. For instance, the tumour origin assessment via deep learning, utilizing cellular graphs within tissues, provides a differential diagnosis for the primary tumour origin using routinely acquired histology slides [10]. It serves as an assistive tool in assigning differential diagnoses to complex cases of metastatic tumors in single-modality scenarios.

In the pursuit of personalized treatments, the limitations of single-modal approaches in capturing the complex heterogeneity of diseases become increasingly apparent. Consequently, the need for effective multi-modal fusion methods has become more prominent. The incorporation of deep learning techniques for the fusion of diverse data types opens new avenues for discovering cancer biomarkers and enhancing clinical decision-making, ultimately aiding in patient stratification and advancing personalized healthcare [11, 12, 13]. Notably, few studies have explored the use of AI for a comprehensive evaluation of recurrence and metastasis risk, incorporating a triad of data sources: molecular data, pathological slides, and clinical information. Acknowledging the transformative potential of AI, this study addresses a significant gap by introducing an innovative AI-driven approach that builds upon existing research. The primary objective is to predict DFS in non-metastatic breast cancer patients, aiming to improve our understanding and prognosis of this disease.

This study contributes to the integration of AI innovation and medical research by developing the novel Deep Learning Clinical Medicine Based Pathological Gene Multimodal (DeepClinMed-PGM) model for DFS prediction. This model effectively integrates molecular data, pathological slides, and clinical information for DFS prediction, representing a significant advancement in employing AI to handle complex tasks such as image analysis and predictive modeling simultaneously. Our approach is in line with current research trends, underscoring the role of AI algorithms in enhancing prognostic accuracy and customization.

## Materials and methods

### Patients and study design

**Figure 1.**
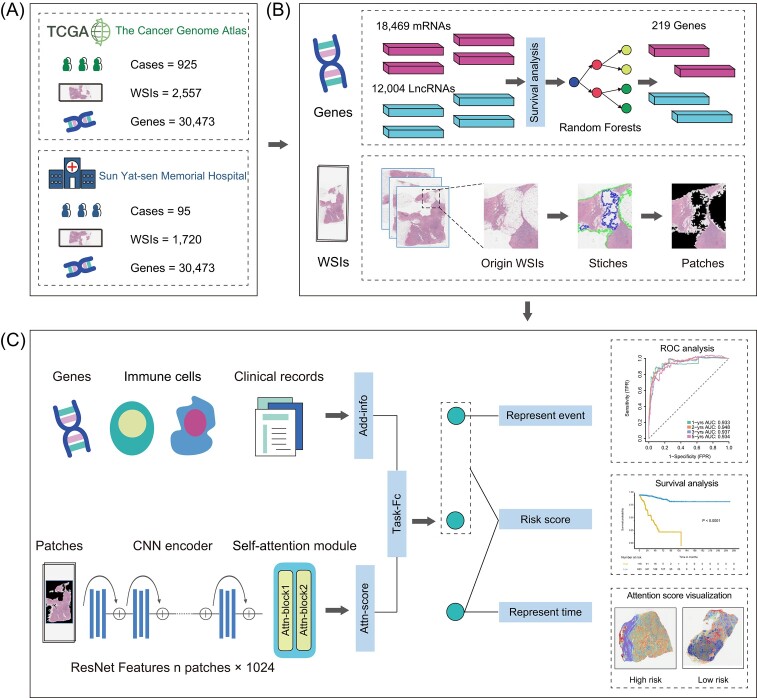
Workflow and graphical methodological overview of this study. (**A**) This project utilized breast cancer data from TCGA as the training set, and data from the SYSMH as the external validation set. (**B**) The data used in this project includes gene data and pathological data. The process of data preprocessing is illustrated. The genetic component was scrutinized through survival disparity analysis to identify mRNA and LncRNA with heightened correlation to survival outcomes. Simultaneously, we conducted segmentation on histopathological slides to remove background regions and partition the patches into smaller blocks. (**C**) Specific model architecture of DeepClinMed-PGM. The filtered genes, along with immune cell characterization and clinical features, were collectively integrated as additional features into the last fully connected layer (Task-fc) of the model. Meanwhile, the histopathological images were directly processed through the backbone model. Following the computation of attention scores, the weighted features of the entire slide were derived. These multimodal fused features were subsequently utilized for predicting patient DFS. In contrast to conventional regression models, this customized model yields three outputs. Two of these outputs represent the probability of patient survival, while the third output signifies the patient's survival time. The final survival score is obtained through their product.

This study design, illustrated in Fig. 1, utilized a comprehensive dataset combining pathology images, molecular data, and clinical information from two sources: The Cancer Genome Atlas (TCGA cohort) and the Sun Yat-sen Memorial Hospital of Sun Yat-sen University (SYSMH) Guangzhou, China. We rigorously applied inclusion and exclusion criteria to select a suitable cohort. Specifically, 1020 patients diagnosed with non-metastatic breast cancer and having preoperative pathology images from 2011 to 2019 were included. Our inclusion criteria were stringent, covering patients with available digital pathology images and necessary clinical data for in-depth analysis. This clinical data included factors like the PAM50 subtype, immune cell data, age, lymph node metastases, tumor size, and clinical T and N stages. Exclusion criteria applied to patients lacking pathology results, those with concurrent malignancies, or issues with pathology images.

The study was structured in several phases, starting with the division of 1020 patients into three cohorts: a training cohort, an internal validation cohort, and an external testing cohort, crucial for automatic pathology tissue segmentation and DFS prediction. A subset of 925 breast cancer patients was further divided, with 741 patients assigned to the training cohort and 184 to the internal validation cohort, both from the TCGA cohort with in total 2557 whole slide images (WSIs) and 30 473 genes. Additionally, 95 breast cancer patients from SYSMH (SYSMH cohort), were selected as the external testing cohort with in total 1720 WSIs and 30 473 genes.

### Pathology images quality enhancement and preprocessing

Prior to surgery, all patients underwent a breast puncture. Our data processing pipeline included both WSIs and molecular features as input. For precision, we scanned all WSIs at a 20× magnification, excluding slides with low resolution, through a KF-PRO-005-EX digital pathological scanner. We collected 4277 hematoxylin and eosin-stained WSIs from the combined cohort of 1020 patients, comprising the TCGA and Hospital (HOSP) cohorts. Notably, the TCGA cohort used typical glass slides fixed in formalin and embedded in paraffin. We implemented an automated WSI layout for each image size, using the Clustering-constrained Attention Multiple Instance Learning (CLAM) collection [14]. After delineating graphical boundaries and removing background color, each WSI was downscaled into 256 × 256 pixel patches at a 20× magnification. Feature extraction was performed using the ResNet50 model, pre-trained on ImageNet, to generate 1024-dimensional feature vectors from each patch [15]. This process utilized a graphical user interface with a batch size of 10, facilitating an efficient feature extraction workflow.

### Transcriptome RNA sequencing preprocessing

To guarantee the caliber and reliability of the molecular data, we standardized the gene expression information for the cohort of 741 patients undergoing training. SYSMH patients had their total RNA procured from Formalin-fixed paraffin-embedded (FFPE) samples by utilizing the QIAGEN FFPE RNeasy kit (QIAGEN GmbH, Hilden, Germany). Analysis of RNA was executed employing the Agilent RNA 6000 Nano Kit (Aglient Technologies, Santa Clara, CA, USA), and the assessment of RNA integrity numbers was carried out to appraise RNA integration through the Agilent Bioanalyzer 2100 (Aglient Technologies, Santa Clara, CA, USA). Total RNA (500 ng) underwent amplification using the Ovation FFPE WTA System (NuGEN, San Carlos, CA, USA), while for fragmentation and labeling we applied the NEBNext® Ultra™ II DNA Library Prep Kit (Illumina). Evaluation of the quality and quantity of amplified libraries was conducted using Qubit (Invitrogen, Carlsbad, CA, USA) and the Agilent Bioanalyzer 2100 (Aglient Technologies, Santa Clara, CA, USA). Subsequent to this, all libraries were subjected to sequencing on a DNBSEQ-T7RS (MGI) with 100 bp paired-end reads. Transformation of base call files into the fastq format was accomplished using cal2Fastq. The normalization of raw data was undertaken utilizing fastp (version 0.20.1) for data processing.

This standardization provided a solid foundation for our computer-based analyses. We then conducted cross-cohort gene screening, identifying 30 473 common genes between the TCGA and SYSMH cohorts. We further employed uni-variate Cox regression analysis within the training cohort, selecting 219 genes significantly associated with prognosis [16]. These genes were chosen based on a stringent significance level of *P* < 0.0116, maintaining high statistical standards. Finally, we applied a random forest analysis, a machine learning technique adept at handling complex molecular data, especially in computer-aided disease prediction [17]. This step increased the robustness of our model.

### Immune cells preprocessing

This study analyzed gene expression data from 96 breast cancer patients at SYSMH to describe the expression profiles of 64 immune and stromal cell types using the XCell tool, a gene set enrichment method capable of quantifying different cell types within tissues [18]. The methodology involved identifying these cell types from patient gene expression data using XCell and integrating this data with clinical information. The aim was to investigate the association between cell type abundance and clinical features of breast cancer.

### Development of the DeepClinMed-PGM model

In this study, we developed a multi-instance learning network that leverages pretherapy pathology images, molecular data, and clinical information to assess risk and predict DFS. The network's architecture comprises two distinct stages: feature extraction and survival prediction. Our approach to tackling the complexities of this task involved a multi-modal data integration strategy within the DeepClinMed-PGM framework. This framework includes essential stages such as quality control, data preprocessing, and the extraction and fusion of features for survival prediction. Employing this advanced deep learning model within the DeepClinMed-PGM framework, our study aims to construct a system capable of providing valuable insights into breast cancer risk assessment and DFS prediction.

In the feature extraction stage, we began by manually partitioning the collection of patches from a single WSI. From these patches, we reduced the feature map obtained from each 256 × 256 × 3 Red-Green-Blue (RGB) image. The patches for multi-instance WSIs in this study relied on a modified ResNet50 model, adapted from the original pretrained ResNet50 architecture. This modified model utilizes 1024-dimensional features output from an earlier convolution layer of the pretrained ResNet50, instead of using the full ResNet50, and incorporates average pooling to improve performance. To efficiently manage the feature-extraction step, we used up to four Graphics Processing Unit (GPUs) running in parallel, each with a batch size of 64.

After automatically extracting features from the WSI patches, we constructed a pathology multi-instance deep learning model to predict slide-based DFS risk scores. This model consists of several components, including feature embedding layers, a self-attention module, and fully connected layers for prediction. We adapted the structure of the feature-embedding layers from a multi-layer perceptron to two linear layers, using the Rectified Linear Unit (ReLU) as the activation function. This change aims to reduce parameter over-fitting during gradient back-propagation. Additionally, the self-attention module was modified to better model the features of WSIs, employing two attention blocks [19]. In the attention-blocks, let $H\ = \ \{ {{{h}_1},\ \ldots ,{{h}_k}} \}$ be a bag of K embeddings, then we propose the following self-attention module:


\begin{eqnarray*}
{\mathrm{z}}\ = \ \mathop \sum \limits_{k = 1}^K {{a}_k}{{{{\bf h}}}_k}
\end{eqnarray*}

where:


\begin{eqnarray*}
{{a}_k}\ = \ \textit{softmax}\left( {{{{{\bf w}}}^{\mathrm{T}}}\left( {\textit{tanh}\left( {{{\bf Vh}}_k^T} \right) \odot \textit{sigm}\left( {{{\bf Uh}}_k^T} \right)} \right)} \right)
\end{eqnarray*}

where ${{\bf w}}\ \in \ {{\mathbb{R}}^L}$ and ${{\bf U}},\ {{\bf V}}\ \in \ {{\mathbb{R}}^{L \times M}}$ are parameters. In our pursuit of improving the efficiency of learning complex relations, our model incorporates two different parallel element-wise non-linearity functions: hyperbolic tangent (tanh()) and sigmoid (sigm()). Including these functions, which cover both negative and positive values, is crucial for ensuring proper gradient flow within the model. We also implemented a mechanism to calculate the attention scores for each patch, achieved by computing the weighted average of the learnable product between the outputs of tanh() and sigm(). To ensure that the attention weights sum to 1, we employed softmax non-linearity. These modifications significantly boost the model's performance, offering robust support for predicting DFS risk scores.

Finally, the attention scores, combined with various clinicopathological characteristics, are integrated using the DeepClinMed-PGM framework to determine the overall multimodal risk score. This multimodal DeepClinMed-PGM risk score is calculated through a deep survival model, influenced by the structure of the Deepsurv model [20]. The input features for this model include a range of parameters, such as the slide-based risk score and clinicopathological characteristics. cTNM staging (T = primary tumor; N = regional lymph node; M = distant metastasis; c = clinical), age at diagnosis, and the expression of immunodeficient cells (e.g. activated B cell, activated CD4 T cell, activated CD8 T cell, and others) are concatenated with the risk score for final score prediction.

### Visualization and interpretation of the model

To thoroughly examine the image areas and features contributing to the network's output, we utilized the GradCAM deep learning algorithm [21] for visualization. This approach, under attention-based learning, automatically visualized attention scores of all tissue regions from the slides in attention heat-maps. These heat-maps enable clinicians to identify tumor regions of high importance. By applying softmax, attention scores were converted into percentages and generated into RGB color-coded heat-maps. Regions with higher attention scores were identified as potential diagnostic tumor tissue, while those with lower scores were considered normal tissue. The CLAM framework eliminated the need for WSI annotation, streamlining the prediction process for important regions.

To understand the critical role of immune cells and immune gene expression in breast cancer and its impact on patient outcomes, we thoroughly analyzed immune cell and gene presence in the TCGA cohort of breast cancer patients. We carefully measured the correlation of 219 related genes and levels of 28 different types of immune cells among the 741 patients in the training group, aiming to uncover differences in immune activity between high-risk and low-risk patients. Additionally, hierarchical clustering analysis was used to visually represent the distribution of these immune cell types and genes among the high-risk and low-risk groups.

To elucidate the functions of these genes, we performed Gene Ontology (GO) and Kyoto Encyclopedia of Genes and Genomes (KEGG) pathway functional enrichment analyses [22, 23]. These analyses categorized gene functions into biological processes, cellular components, and molecular functions. Furthermore, we evaluated the abundance of tumor-infiltrating immune cells in the training cohort (*n* = 741), acknowledging their vital role in prognosis prediction and lymph node pathology detection. Notable differences in immune cell infiltration between high-risk and low-risk groups were observed, providing insights into the underlying mechanisms of breast cancer progression.

### Implementation details

Firstly, we define the data root directory, which is the location storing all training and validation data. The maximum number of epochs for model training is specified by the *max_epochs* parameter, with a default of 30 epochs. The learning rate is set to 1e-4, and the weight decay defaults to 1e-5, both of which jointly affect the convergence speed and final performance of the model. To ensure reproducibility of experimental results, we control randomness by setting the random seed to 1. The number of folds for cross-validation is specified by the *k* parameter, with a default of 10 folds. If training needs to start or end from a specific fold, the *k_start* and *k_end* parameters can be used, with defaults of −1, indicating starting from the last fold and ending at the first fold, we set it to 1 to 2 in this experiment.

The results directory is used to store all experimental results, defaulting to the results folder in the current working directory. We also provide the *split_dir* parameter, allowing users to manually specify split sets rather than inferring from task and label score parameters. To better monitor the training process, TensorBoard logging is enabled through the *log_data* parameter, which is off by default. The testing parameter serves as a debugging tool for development and testing stages, defaulting to off. Early stopping is an optional feature enabled by setting the parameter to *True*, which is off by default.

The choice of optimizer is controlled by the *opt* parameter, offering two options: Adam and SGD (Stochastic Gradient Descent), with Adam as the default choice. We also introduce dropout mechanism, enabled by the *drop_out* parameter, with a default dropout rate of 0.25. Experiment code is used to save and identify different experimental settings. Additionally, we provide the ‘weighted_sample' parameter to enable weighted sampling, and ‘gene' and ‘cli' parameters to control the usage of gene and clinical data, respectively. Finally, the task parameter specifies the task type, with DFS task chosen in this study.

### Quantification and statistical analysis

In our study, we conducted survival analysis using the Kaplan–Meier (KM) method alongside the log-rank test, allowing for the assessment and comparison of survival outcomes among different patient groups. Hazard ratios (HRs) and their corresponding 95% confidence intervals (CIs) were determined through Cox regression analysis, shedding light on the factors impacting patient outcomes. To stratify patients into high- and low-risk groups, we utilized optimal cutoff values, identified using the R package survminer. This stratification was essential for evaluating the impact of the signatures generated on DFS. The prognostic or predictive accuracy of these signatures was assessed through receiver operating characteristic curve (ROC) analysis. Sensitivity and specificity were evaluated using the area under the ROC curve (AUC), which serves as an effective metric for assessing the predictive performance of the signatures for DFS. Additionally, to gauge the clinical utility of our prediction model, we conducted decision curve analysis (DCA), as detailed in [24]. DCA is instrumental in determining the model's practical value in clinical decision-making.

For the technical aspects, we utilized the NCCS-GZ Guangzhou National Supercomputer Platform and NVIDIA Tesla K80 GPU to support the functions of CLAM packet 15. WSI processing and feature extraction were conducted using Python (version 3.7.7). For WSI processing and segmentation, we used Column (version 7.0.0) and Opencv-Python (version 4.1.1). The Python deep learning library (version 1.5.1) was implemented to train deep learning models on GPUs. The torchvision (versions 1.12.1 and 0.1.8) was employed to load ResNet50 and extract features. For plotting and numerical vectors calculation, we used the Matplotlib library (version 3.1.1) and numpy library (1.18.1), respectively. Attention scores visualization was performed using basemap (version 1.1.0). Data analysis was conducted using Pandas (version 1.0.3) and Scipy (version 1.3.1). Various sub-indexes were calculated using the Scikit-learn scientific computing manual (version 0.22.1). Other statistical analyses were carried out using R version 4.1.2 (https://www.r-project.org). A *P* value < 0.05 was considered statistically significant.

## Results

### Patient characteristics

This retrospective cohort study included a total of 1020 non-metastatic breast cancer patients, comprising 925 patients from the TCGA cohort, with a median age of 57 years [interquartile range (IQR): 48–66 years], and 95 patients from the HOSP cohort, with a median age of 50 years (IQR: 44–57.5 years). The patient enrollment process is visually depicted in Fig. 1A, providing a clear overview of the study's patient selection methodology. To ensure a thorough analysis, patients were strategically divided into distinct cohorts: the training cohort (*n* = 741), the internal validation cohort (*n* = 184), and the external testing cohort (*n* = 95). Detailed information on the clinicopathological characteristics and treatment outcomes of these patients is presented in Table 1 .

**Table 1.** Clinical characteristics of patients in the TCGA and SYSMH cohorts.

Characteristic	No. (%)			
	All patients (*n* = 1020)	TCGA cohort (*n* = 925)		SYSMH cohort (*n* = 95) External test cohort (*n* = 95)
		Training cohort (*n* = 741)	Internal validation cohort (*n* = 184)	
**Age, median (IQR), years**	57 (48–66)	59 (49–67)	56 (48–66)	50 (44–57.5)
**Clinical T stage**				
1	295 (28.92%)	198 (26.72%)	46 (25.00%)	51 (53.58%)
2	587 (57.55%)	431 (58.16%)	115 (62.50%)	41 (43.16%)
3	115 (11.27%)	94 (12.69%)	18 (9.78%)	3 (3.16%)
4	23 (2.25%)	18 (2.43%)	5 (2.72%)	0 (0%)
**Clinical N stage**				
1	508 (49.80%)	377 (50.88%)	86 (46.74%)	45 (47.37%)
2	326 (31.96%)	246 (33.20%)	57 (30.98%)	23 (24.21%)
3	116 (11.37%)	78 (10.53%)	24 (13.04%)	14 (14.74%)
4	70 (6.86%)	40 (5.40%)	17 (9.24%)	13 (13.68%)
**Clinical TNM stage**				
1	204 (20.00%)	140 (18.89%)	33 (17.93%)	31 (32.63%)
2	578 (56.67%)	441 (59.51%)	101 (54.89%)	36 (37.89%)
3	238 (23.33%)	160 (21.59%)	50 (27.17%)	28 (29.57%)

In terms of clinical staging, the percentage of patients with pathological clinical TNM stage III was 22.7% in the training and internal validation cohorts from the TCGA, and 29.57% in the external testing cohort from the hospital. The median follow-up times were 59 months (IQR 49–67) for the training cohort, 56 months (IQR 48–66) for the internal validation cohort, and 50 months (IQR 44–57.5) for the external testing cohort.

### Proposed pathology-based deep learning model

In our endeavor to utilize advanced deep-learning models for predicting DFS based on WSIs, we implemented a pathology-based integration approach using the DeepClinMed-PGM Multi-Modality Model framework. The structure of this framework is depicted in Fig. 1 and supplementary Fig. S1 (see online supplementary material).

The initial step involved randomly dividing the TCGA cohort of 925 patients into training and internal validation sets in an 8 : 2 ratio. This division was crucial for automated preoperative pathology tissue segmentation. The architecture for processing pathology images is comprehensively outlined in Fig. 1B, offering a specific blueprint for primary tissue segmentation. This process operates on individual WSIs, utilizing a modified CLAM architecture and an unlabeled bag of patches corresponding to the primary tumor tissue area for training.

Following the automated extraction of the primary tumor's patches from pathology images, we developed a deep pathology-based multi-instance learning survival model to predict a patient-based risk score for DFS. Using the modified ResNet50 architecture for feature extraction and a transfer learning algorithm, this model effectively identifies critical features from the extracted patches (Fig. 1B). These features are then refined through attention gates to create a distinctive feature vector for each patient. The fully connected layers utilize this vector to compute the patient-based DFS risk score, an essential metric for guiding treatment decisions and monitoring patient progress. We further integrated molecular and clinicopathological features with the pathology-based survival model to form the DeepClinMed-PGM. This integration amalgamates attentive slide scores, molecular, and clinicopathological data, providing a personalized approach to DFS prediction (Fig. 1C).

### Predictive performance through a deep learning model based on pathology data

After training our network, we applied a modified multi-instance deep neural network to conduct an in-depth analysis of preoperative pathology slides for predicting DFS. The deep learning pathology multi-instance learning model, known as the pathology-based survival model, exhibited a high level of predictive accuracy for DFS (supplementary Figs. S2, S3, and S4, see online supplementary material). Time-dependent ROC analysis provided valuable insights into the model's performance. In the training cohort, the AUC values were 0.790, 0.777, and 0.783 for 1-, 3-, and 5-year DFS predictions, respectively, indicating the model's effectiveness in forecasting DFS within this group (Fig. S3).

In the internal validation cohort, the corresponding AUC values were 0.725, 0.834, and 0.852, further highlighting the model's predictive strength across different timeframes. In external testing cohort 1, the pathology-based survival model demonstrated AUC values of 0.87, 0.82, and 0.77 for 1-, 2-, and 3-year DFS predictions, respectively. These results underscore the model's reliability and applicability across various external testing environments. The concordance index (C-index) for the deep learning model in DFS prediction was consistent, recording 0.749, 0.804, and 0.777 in the training cohort, internal validation cohort, and external testing cohort, respectively.

By stratifying patients in the training cohort according to their predicted survival scores, we significantly enhanced breast cancer risk assessment. There was a notable difference in DFS between patients with low- and high-risk scores, indicated by a HR of 0.224 (95% CI 0.138–0.363, *P* < 0.001; Fig. S3A). This trend was consistently observed in the internal validation cohort, with an HR of 0.072 (95% CI 0.016–0.324, *P* < 0.001; Fig. S3B), and corroborated in an external validation cohort, with an HR of 0.343 (95% CI 0.124–0.950, *P* < 0.001; Fig. S3C). These findings reaffirm the strong correlation between predicted survival scores and DFS outcomes.

### Enhancing prediction through multimodal data integration and clinical implication

In our study, we incorporated clinicopathological characteristics related to DFS in both univariate and multi-variable Cox regression analyses. Our findings showed that even after adjusting for various clinicopathological variables, including clinical TNM stage, the deep learning pathology-based survival model remained an independent prognostic factor for DFS within the training cohort, as detailed in supplementary Fig. S5, see online supplementary material.

Aiming to enhance prediction accuracy and highlight the importance of multi-modal data integration, we focused on variables such as 219 genes and clinicopathological factors like PAM50 subtypes, age, clinical TNM staging, and immune cell data (e.g. activated B cells, activated CD4 T cells, activated CD8 T cells). These elements are known to significantly affect the biological behavior and prognosis of breast cancer [2, 3]. To achieve a more precise and clinically relevant approach for evaluating DFS prediction, we integrated these 32 clinicopathological characteristics with the deep learning model, resulting in the development of the DeepClinMed-PGM model. DeepClinMed-PGM synergizes attentive slide scores with clinicopathological data, enabling personalized DFS predictions. This methodology significantly enhances prediction accuracy and augments the clinical relevance of assessing an individual's risk of disease recurrence.

**Figure 2.**
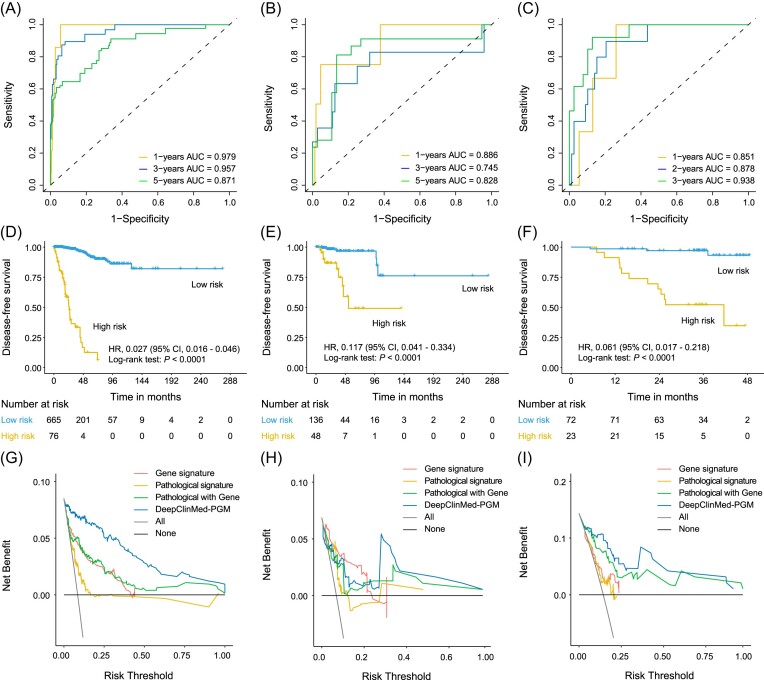
AUC and KM curves for training sets, validation sets, and test sets. (**A**) Training set ROC, (**B**) validation set ROC, (**C**) test set ROC, (**D**) training set KM curve, (**E**) validation set KM curve, and (**F**) test set KM curve. (**G**) Decision curve of traning set. (**H**) Decision curve of validation set. (**I**) Decision curve of test set.

After integrating multimodal data into the DeepClinMed-PGM model, there was a marked improvement in its predictive performance for DFS. Within the training cohort (Fig. 2A), the AUC values for 1-, 3-, and 5-year DFS prediction rose to 0.979, 0.957, and 0.871, respectively. In the internal validation cohort, the AUC values were 0.886, 0.745, and 0.825 (Fig. 2B), and in the external testing cohorts, the AUC values reached 0.851, 0.878, and 0.938 for 1-, 2-, and 3-year DFS prediction (Fig. 2C). Moreover, the DeepClinMed-PGM model demonstrated C-index values of 0.925, 0.823, and 0.864 within the training cohort, internal validation cohort, and external testing cohort, respectively. These results indicate a superior predictive accuracy compared to the standalone deep learning pathology-based survival model for survival prediction. Detailed information for 1-, 2-, and 3-year DFS prediction and treatment outcomes of these patients is presented in Table 2.

**Table 2.** Diagnostic accuracy of prognostic characteristics for patients in training set.

Characteristic	1-year AUC (95% CI)	3-year AUC (95% CI)	5-year AUC (95% CI)
**Age**			
**≤ 65 years**	0.985 (0.973–0.997)	0.951 (0.914–0.988)	0.871 (0.803–0.939)
**> 65 years**	0.953 (0.918–0.988)	0.973 (0.932–1)	0.868 (0.702–1)
**Sex**			
**Female**	0.978 (0.966–0.991)	0.957 (0.927–0.987)	0.87 (0.806–0.935)
**Clinical T stage**			
**T1**	0.997 (0.991–1)	0.912 (0.818–1)	0.775 (0.593–0.958)
**T2**	0.984 (0.972–0.997)	0.981 (0.964–0.999)	0.899 (0.814–0.984)
**T3**	0.927 (0.846–1)	0.889 (0.724–1)	0.933 (0.836–1)
**T4**	0.909 (0.734–1)	1	1
**Clinical N stage**			
**N0**	0.991 (0.981–1)	0.976 (0.945–1)	0.872 (0.781–0.963)
**N1**	0.962 (0.933–0.991)	0.966 (0.918–1)	0.875 (0.763–0.986)
**N2**	1 (1–1)	0.855 (0.669–1)	0.927 (0.826–1)
**N3**	0.939 (0.857–1)	0.946 (0.83–1)	0.906 (0.691–1)
**Clinical M stage**			
**M0**	0.983 (0.972–0.994)	0.956 (0.922–0.991)	0.859 (0.786–0.931)
**Clinical TNM stage**			
**Stage 1**	0.995 (0.984–1)	0.992 (0.973–1)	0.677 (0.423–0.93)
**Stage 2**	0.988 (0.978–0.999)	0.979 (0.96–0.998)	0.898 (0.812–0.983)
**Stage 3**	0.939 (0.887–0.99)	0.895 (0.788–1)	0.891 (0.796–0.986)
**Molecular subtype**			
**Basal**	0.945 (0.901–0.989)	0.921 (0.84–1)	0.938 (0.867–1)
**HER2**	1	1	1
**Luminal A**	0.991 (0.979–1)	0.946 (0.858–1)	0.734 (0.555–0.914)
**Luminal B**	0.959 (0.924–0.994)	0.984 (0.959–1)	0.96 (0.909–1)
**Triple-negative**	0.955 (0.918–0.991)	0.929 (0.854–1)	0.945 (0.883–1)
**Non-triple-negative**	0.98 (0.966–0.994)	0.971 (0.934–1)	0.853 (0.76–0.946)
**Normal**	NA (Not applicable)	1	1

Our DeepClinMed-PGM model demonstrated significant distinctions in DFS between patients with high- and low-risk scores. By employing an optimized cutoff value of 16.135, derived through the DeepClinMed-PGM model, we stratified patients into high-risk and low-risk groups. The DeepClinMed-PGM model's robust discriminative capabilities were consistently evident across various cohorts. In the training cohort, it exhibited an HR of 0.027 (95% CI 0.0016–0.046, *P* < 0.0001) (Fig. 2D), in the internal validation cohort an HR of 0.117 (95% CI 0.041–0.334, *P* < 0.0001) (Fig. 2E), and in the external cohorts an HR of 0.061 (95% CI 0.017–0.218, *P* < 0.0001) (Fig. 2F). Furthermore, the DeepClinMed-PGM model's versatility extended to its ability to predict recurrence risks and differentiate between high- and low-risk patients, even when considering factors such as age at diagnosis, sex, and clinical TNM stage of breast cancer, with all results showing statistical significance (all *P* < 0.001) (see supplementary Fig. S5).

Additionally, our DCA indicated that the DeepClinMed-PGM model consistently offered a superior net benefit compared to the pathology-based survival model, gene signature, and pathological signature alone, across a wide range of threshold probabilities in all cohorts (see Fig. 2G, H, I). This finding highlights the substantial clinical value of the DeepClinMed-PGM model in early DFS prediction, surpassing the individual contributions of the pathology-based survival model, gene signature, and pathological signature. Clinicians can leverage this integrated system by combining pathology images, molecular data, and clinicopathological information to provide personalized DFS predictions. This comprehensive approach aids in identifying risks of recurrence and metastasis, and assists in developing customized treatment strategies.

### Visualization and interpretation

**Figure 3.**
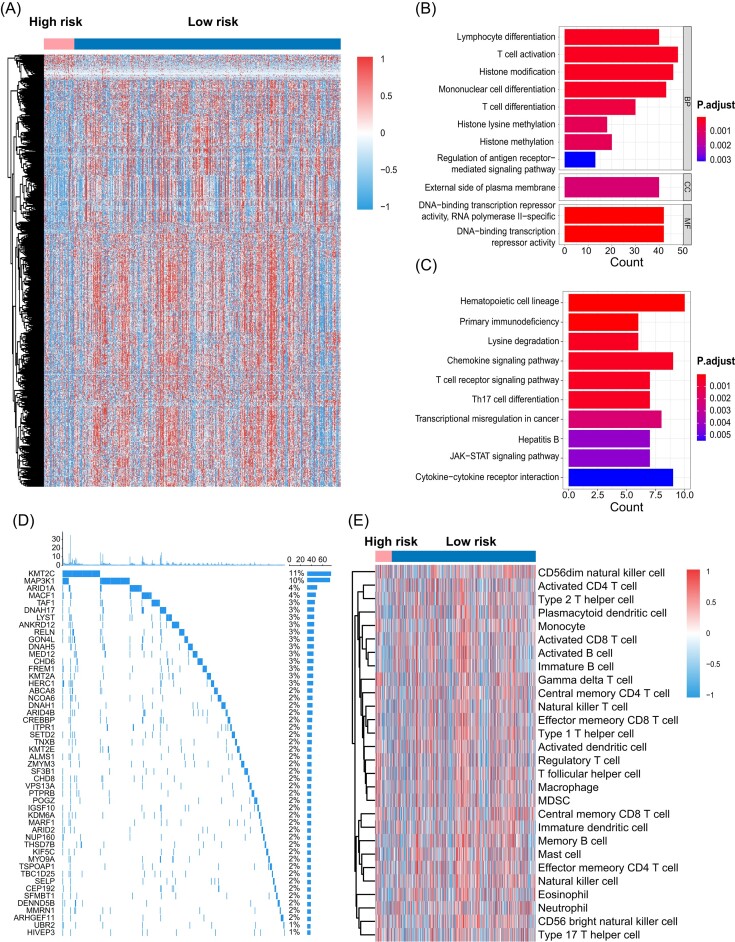
Analysis of difference between high and low expression groups. (**A**) Differential genes in high- and low-risk groups. (**B**) and (**C**) GO and KEGG pathway analysis in low-risk group. (**D**) Mutation landscape in low-risk group. (**E**) Immune cell infiltration analysis in high- and low-risk groups.

Our research delved into the correlation between high-density regions within tumor hotspots and the tumor micro-environment, emphasizing the significant prognostic information provided by imaging techniques. Focusing on an objective evaluation, we utilized WSIs from pathological samples for our analysis. A comprehensive examination, including RNA sequencing in the training cohort, identified 219 differentially expressed genes between the high-risk and low-risk groups (Fig. 3A). The subsequent GO enrichment analyses highlighted critical pathways related to immunity and transcription, such as T-cell activation, monocyte differentiation, and histone modification. These pathways play a vital role in regulating gene expression in breast cancer cells and significantly impact the progression of the disease.

Moreover, KEGG analysis indicated enrichment in pathways like Th17 cell differentiation, the T cell receptor signaling pathway, and chemokine signaling. Within the training cohort, our analysis showed that KMT2C and MAP3K1 had high mutation frequencies, at 11% and 10% respectively, as illustrated in Fig. 3D. KMT2C mutations are frequently observed in metastatic breast cancers that have hormone receptors and low levels of HER2 [25–27]. Additionally, KMT2C mutations and deletions are observed in pancreatic ductal adenocarcinoma [28], with reduced KMT2C expression in biopsies correlating with improved clinical outcomes. As a histone modifier, KMT2C also frequently mutates in esophageal squamous cell carcinoma [29]. MAP3K1, conversely, is involved in pathways regulating cell proliferation and apoptosis [30, 31]. Our investigation into immune cell infiltration in the tumor revealed notable differences between high-risk and low-risk groups, crucial for prognosis prediction and lymph node pathology detection, as shown in Fig. 3E. These findings highlight the importance of high-density regions within the tumor microenvironment, offering insights into distinct molecular and immune signatures associated with different risk groups.

In our analysis, we focused on image regions and influential features impacting the output of the pathology-based survival model. This endeavor aimed to provide clinicians with a deeper understanding of the network's predictions, enhancing their knowledge about tumor zones. To examine the potential correlation between prediction capacity and deep learning features in WSIs, we employed the GradCAM deep learning algorithm. This technique enabled us to convert attention feature maps into visually informative heatmaps, offering an interpretable view of the model's focus areas.

**Figure 4.**
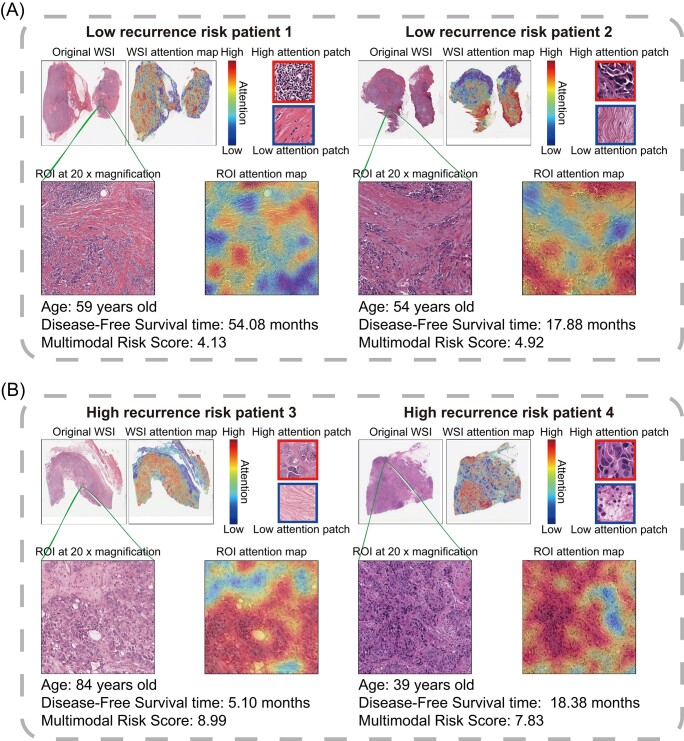
Pathological heatmaps show that AI identifies areas of heightened response in region of interest (ROI). (**A**) AI-identified area heat maps for patients at low risk of recurrence. (**B**) AI-identified area heat maps for patients at high risk of recurrence.

The heatmaps generated in our study vividly highlighted areas of heightened response within the tumor tissues, using warm colors (e.g. red) to indicate high influence and cool colors (e.g. blue) for lower influence on the model's predictions. Darker shades signified a stronger network response with higher attention weights, indicating an intensified focus of the model on those specific areas. The attention maps in shades of blue primarily depicted structural aspects of the tumors, such as boundaries, shapes, and textures. In contrast, red-biased attention maps predominantly captured high-level semantic tumor traits derived from anatomical images, along with functional insights. For instance, Fig. 4 presents examples for four patients, where those with a high risk of recurrence or metastasis showed concentrated hotspots both near and distant from the tumor, whereas patients without such risks exhibited focal concentration mainly within the tumor region. These patterns, recurring across most heat maps, suggest that concentrated hotspots within the tumor region may correlate with improved DFS.

**Figure 5.**
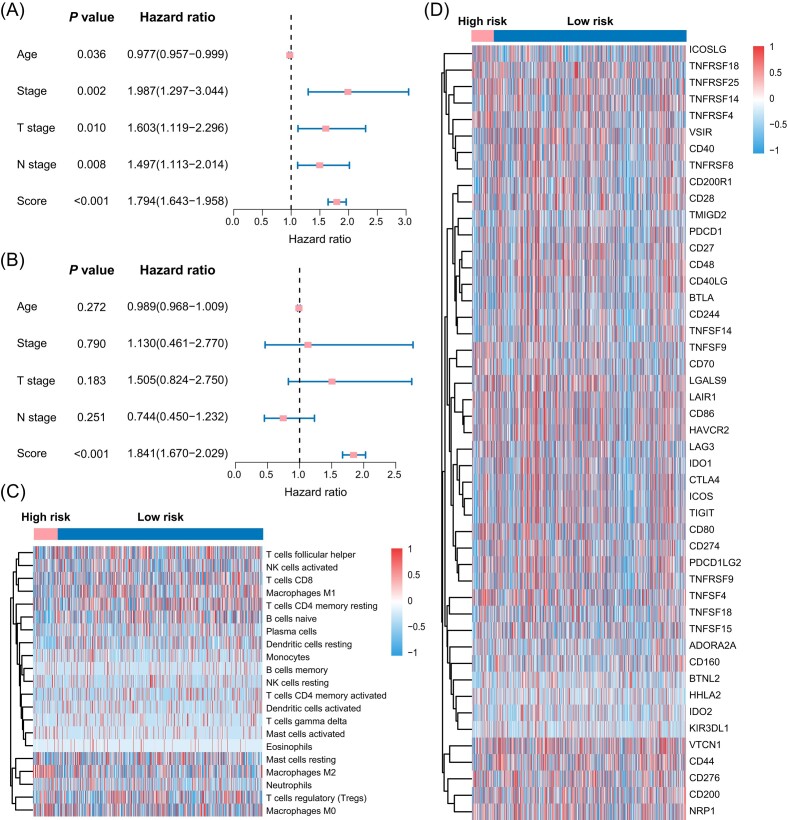
Validation of differential molecular characteristics among different risk groups. (**A**) Univariate Cox analysis. (**B**) Multivariate Cox analysis. (**C**) Differences in immune infiltration between high- and low-risk groups. (**D**) Differences in immune checkpoint expression between low- and high-risk groups.

To assess the predictive role of clinical characteristics on DFS in breast cancer patients, we conducted univariate regression analyses in the training and test groups. These analyses evaluated whether the risk score could remain an independent predictor when considering other conventional clinical variables. The results indicated that clinical characteristics like age, stage, T stage, and N stage could not independently predict DFS in breast cancer patients, whereas the risk score was a more effective predictor in both the training and test groups (*P* < 0.001). The immune cells were annotated using the CIBERSORT algorithm, and subsequent immune infiltration analysis revealed higher infiltration of macrophages M2 and M0 in the high-risk group (Fig. 5C). Additionally, our examination of immune checkpoint gene expression in high- and low-risk groups showed generally higher expression in the low-risk group.

**Figure 6.**
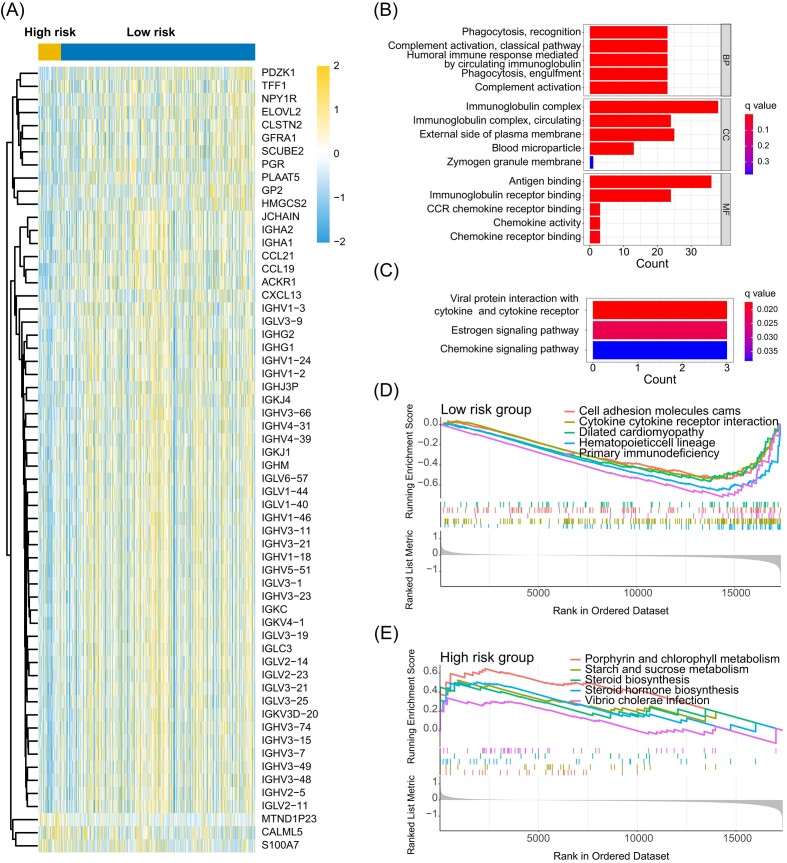
Immunological analysis of differential molecular characteristics among different risk groups. (**A**) Differences in immune factor expression between high- and low-risk groups. (**B, C**) GO and KEGG pathway analysis in high-risk group. (**D, E**) Pathway differences between high- and low-expression groups were analyzed by GSEA.

Further, the expression of immune factor-related genes was also higher in the low-risk group. Our GO analysis of immune factor-associated genes indicated their enrichment primarily in complement activation and phagocytosis-associated pathways, mainly located in the immunoglobulin complex (Fig. 6B). Concurrently, KEGG pathway analysis revealed significant enrichment in the estrogen signaling pathway and chemokine signaling pathway (Fig. 6C). Additionally, Gene Set Enrichment Analysis (GSEA) in Fig. 6D highlighted enrichment in cell adhesion molecules and cytokine–cytokine receptor interactions in the low-risk group. In contrast, the high-risk group showed enrichment in pathways related to starch and sucrose metabolism, as well as steroid biosynthesis.

In conclusion, our findings emphasize the effectiveness of the risk score in predicting DFS in breast cancer and illuminate the distinct molecular and immunological profiles characteristic of different risk groups.

## Discussion

Our study introduced and evaluated the novel DeepClinMed-PGM model, a pathology-based multi-instance deep learning model utilizing multimodal data for accurately predicting DFS in breast cancer patients. This model's predictive accuracy significantly improves risk stratification, aiding clinicians in formulating precise treatment strategies and follow-up plans. Visualization techniques employed in our study offered deeper insights into the decision-making process, correlating predictions with the tumor microenvironment, and presented a transformative approach for enhanced breast cancer prognosis and personalized treatment strategies. The results underscore the potential of advanced computational techniques in refining prognostic predictions and individualized treatment planning.

The rapid advancement in AI technology has made the application of multi-modal deep learning methods increasingly viable in aiding breast cancer prognosis and treatment planning. Deep learning models have proven their utility as valuable prognostic tools, supporting oncologists in tailoring patient management strategies. For instance, these models can predict RNA-Seq tumor expression and interpret diverse molecular phenotypes from densely mapped cancer pathology slides, all derived from WSIs [32, 33]. However, relying solely on single-modality medical imaging data often falls short in providing comprehensive information for precise breast cancer diagnosis. The diagnosis of breast cancer involves various medical data sources, including pathology, genetic molecular data, mammography, ultrasound, magnetic resonance imaging (MRI), and clinical text. The complexity inherent in these diverse data sources makes comprehensive analysis and accurate diagnosis more challenging.

Therefore, the unified processing of multimodal inputs can streamline patient triage and enhance the clinical decision-making process. The future of breast cancer diagnosis and treatment planning will undoubtedly rely on the integration of a broad spectrum of clinical knowledge, combining pathological features, clinical guidelines, and the expertise of medical professionals. Integrating multimodal data derived from histopathological, molecular, and clinicogenomic machine-learning models promises to improve patient risk stratification and deepen our understanding of treatment responses in cancer [34, 35].

Our study not only reinforces the generalization of our approach but also lays a solid foundation for its validation. The innovation of our research lies in developing the novel AI-driven DeepClinMed-PGM model. This model reflects the ongoing shift towards utilizing AI algorithms for complex tasks in the medical field. While deep learning models in the medical domain have gained considerable attention, few have seamlessly integrated histopathological data, molecular data, and clinicogenomic features into a genuinely multi-modal model [36, 37, 38]. Our approach aligns with the increasing recognition of the complex interplay between biological attributes and clinical factors in cancer prognosis. It culminates in an AI-assisted model that equips clinicians with AI-driven insights, thereby enabling more informed decision-making in breast cancer management.

In the foreseeable future, deep learning algorithms are poised to significantly advance the field of molecular functional visualization studies using multi-modal data, particularly in elucidating pathogenic pathways associated with breast cancer. Molecular functional visualization studies are crucial not only for understanding the pathological mechanisms of breast cancer but also for offering novel insights and methodologies for clinical application [39, 40]. Specifically, deep learning models have been developed to predict RNA-Seq tumor expression, and human-interpretable features from densely mapped cancer pathology slides extracted from WSIs are used to forecast a range of molecular phenotypes [41, 42]. Our research aligns with previous studies emphasizing the pivotal role of medical imaging, especially pathology images, in enhancing our understanding of breast cancer characteristics, consistent with previous research that has highlighted the value of WSIs and molecular data in exploring tumor evolution and gaining detailed insights into tumor morphology and the surrounding micro-environment.

Despite promising outcomes, several inherent limitations of our study need careful consideration. The use of large retrospective datasets and the retrospective design introduce inherent biases and the potential for confounding factors. Moreover, the applicability of our developed models to diverse populations and varied clinical settings requires further validation and scrutiny. Additionally, the issue of interpretability in AI models remains a central point of discussion in research, highlighting the need for methodologies that enhance transparency and elucidation.

Our multi-modal deep learning model initiatives are expected to serve various purposes, including identifying novel anti-tumor gene expression patterns, exploring the distribution of immune cells, facilitating the deployment of innovative effector immune cells, and generating molecular entities for future clinical investigations. By comprehensively integrating diverse medical data modalities and clinical knowledge, this AI model is set to significantly enhance early diagnosis, treatment effectiveness, and prognosis prediction in breast cancer. Ultimately, it will provide healthcare practitioners with more reliable and interpretable diagnostic results, leading to improved treatment outcomes and survival rates among breast cancer patients.

## Conclusion

The multi-instance risk oncology assessment via deep-learning multi-modality model introduced in our study represents a novel and effective approach for DFS prediction in non-metastatic breast cancer patients. By integrating multi-modal data, the model demonstrates enhanced predictive accuracy. This advancement underscores the potential of such models in aiding clinical decision-making and facilitating personalized treatment strategies for breast cancer patients. As the field of AI continues to progress, our research contributes to the foundation for further exploration in this domain. It opens avenues for refining models to increase their precision, elucidating the interpretability of complex AI systems, and expanding their application to a broader range of cancers.

## Supplementary Material

pbae012_Supplemental_File

## Data Availability

All data reported in this paper will be shared by the lead contact upon request. This paper does not report original code. Any additional information required to reanalyze the data reported in this paper is available from the lead contact upon request.
