# Supplementary material for: Deep learning-based multi-modal data integration enhancing breast cancer disease-free survival prediction
Source: Precis Clin Med. 2024 May 29;7(2):pbae012. doi: 10.1093/pcmedi/pbae012 (PMC11190375; doi:10.1093/pcmedi/pbae012)
Supplement: pbae012_Supplemental_File [file pbae012_supplemental_file.pdf]

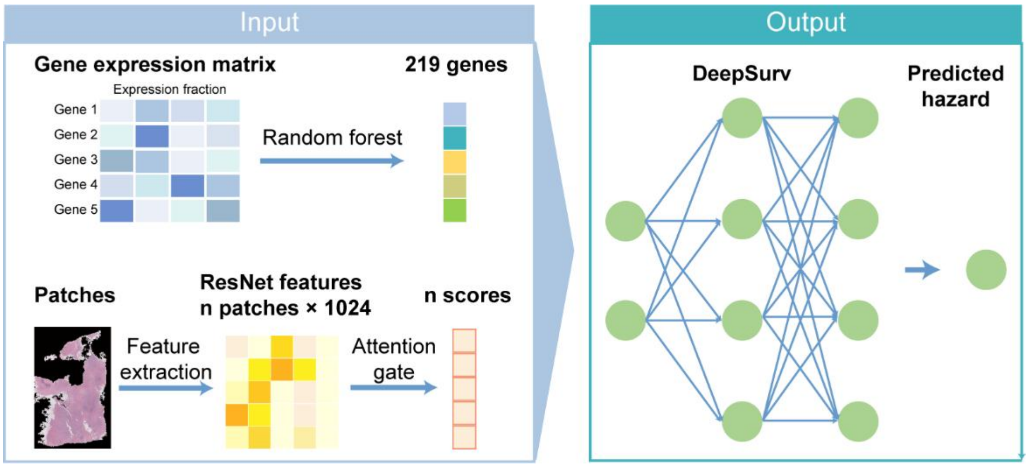

Figure S1. Algorithm flowchart of DeepClinMed model for RNA and WSIs.

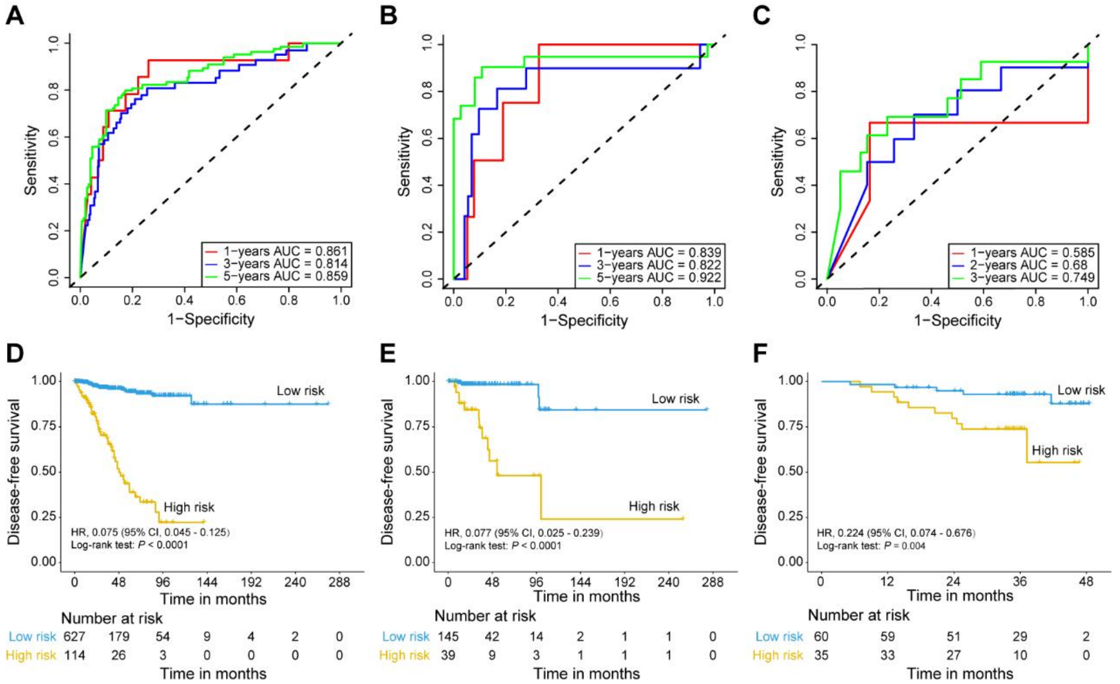

Figure S2. AUC curves and survival curves for training sets, validation sets and test sets.

(A) training set ROC, (B) validation set ROC. (C) test set ROC. (D) training set KM curve. (E) validation set KM curve. (F) test set KM curve.

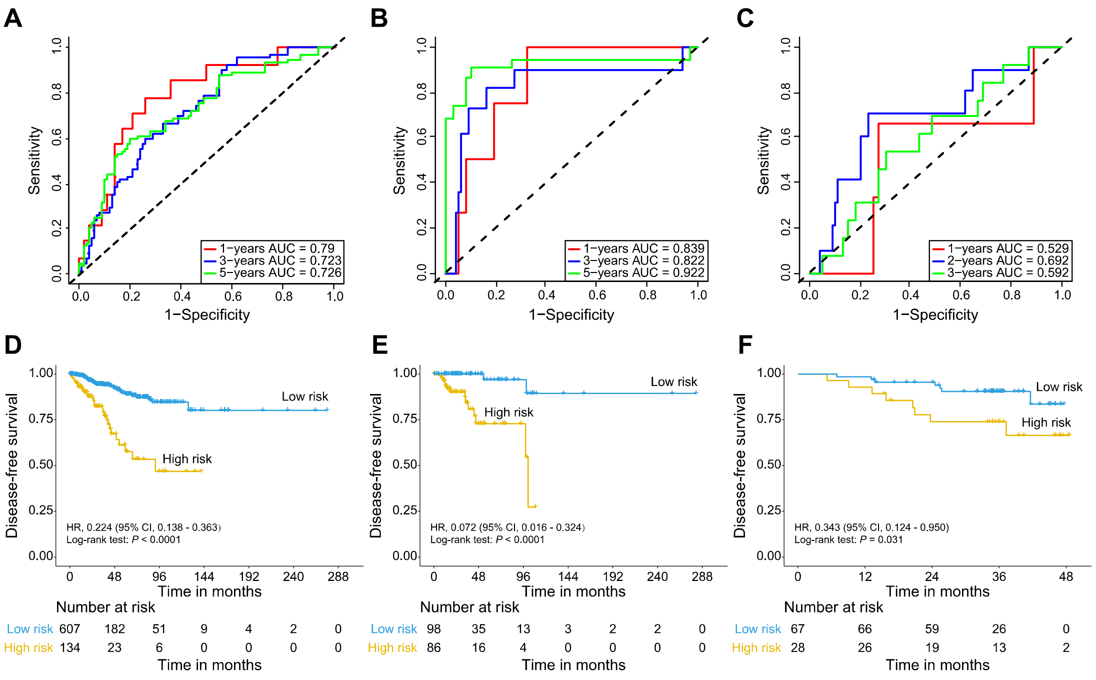

Figure S3. AUC curves and survival curves for training sets, validation sets and test sets. (A) training set ROC, (B) validation set ROC. (C) test set ROC. (D) training set KM curve. (E) validation set KM curve. (F) test set KM curve.

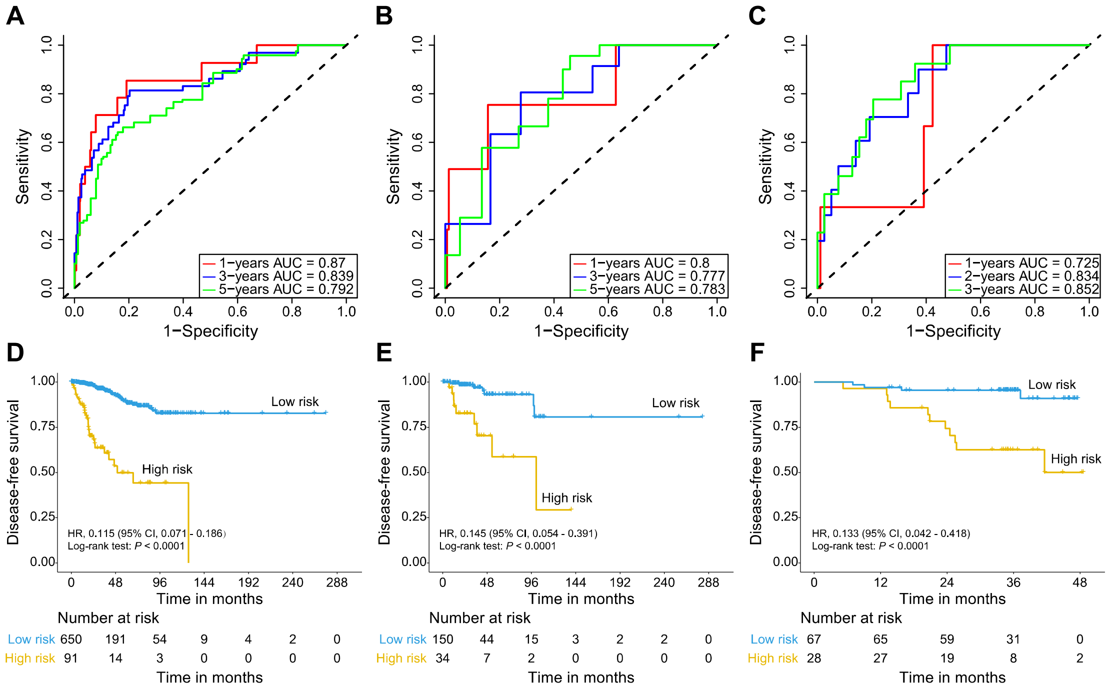

Figure S4. AUC curves and survival curves for training sets, validation sets and test sets. (A) training set ROC, (B) validation set ROC. (C) test set ROC. (D) training set KM curve. (E) validation set KM curve. (F) test set KM curve.

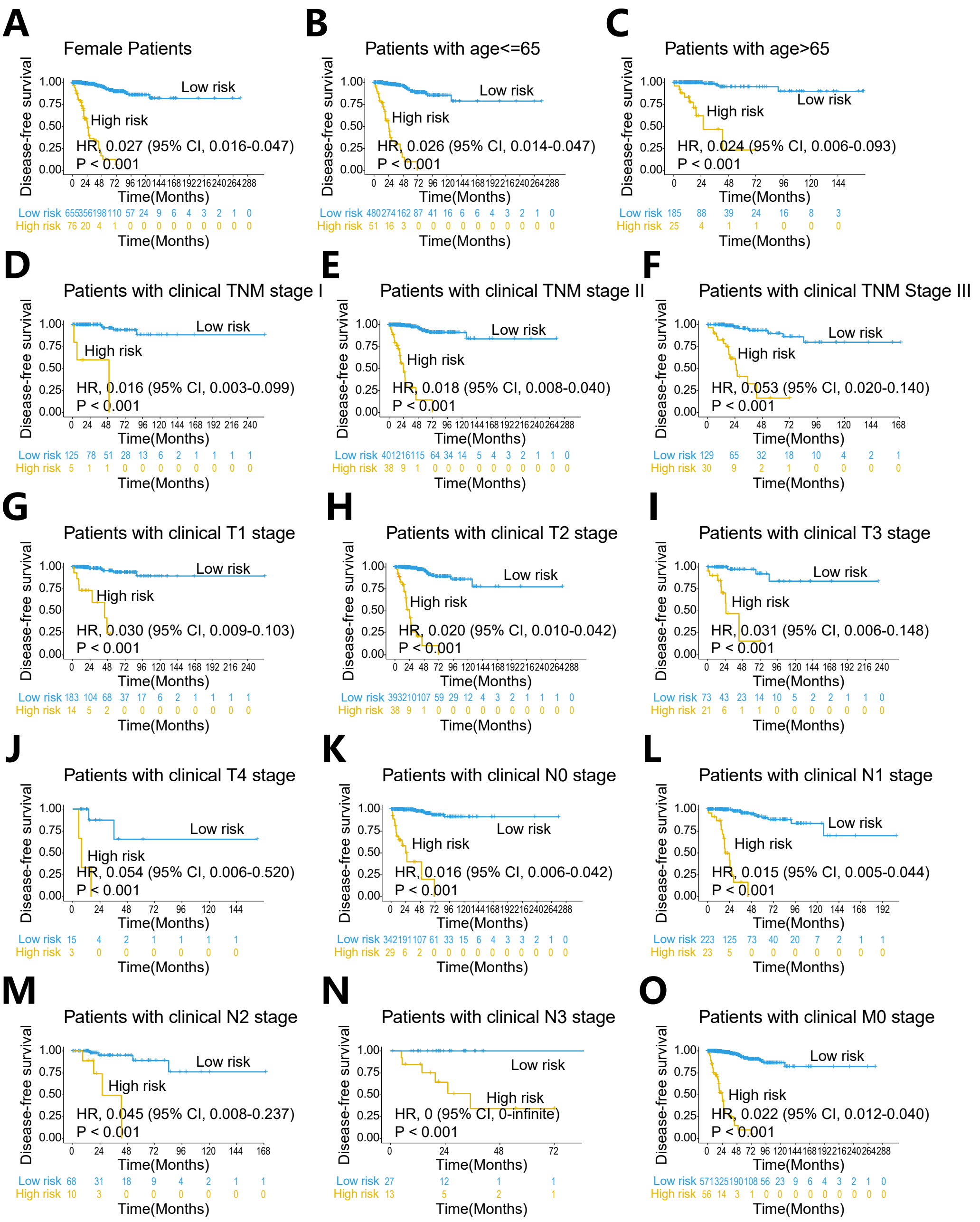

Figure S5 Survival curves for subgroup analysis. (A) Female Patients, (B) Patients with age<=65. (C) Patients with age>65. (D) Patients with clinical TNM stage I. (E) Patients with clinical TNM stage II (F) Patients with clinical TNM Stage III. (G) Patients with clinical T1 stage (H) Patients with clinical T2 stage. (I) Patients with clinical T3 stage. (J) Patients with clinical T4 stage. (K) Patients with clinical N0 stage. (L) Patients with clinical N1 stage. (M) Patients with clinical N2 stage. (N) Patients with clinical N3 stage. (O) Patients with clinical M0 stage.
